# Biomimetic nanocarriers: integrating natural functions for advanced therapeutic applications

**DOI:** 10.3762/bjnano.15.127

**Published:** 2024-12-16

**Authors:** Hugo Felix Perini, Beatriz Sodré Matos, Carlo José Freire de Oliveira, Marcos Vinicius da Silva

**Affiliations:** 1 Department of Immunology, Microbiology and Parasitology. Biological and Natural Sciences Institute. Federal University of Triângulo Mineiro. Uberaba, Minas Gerais, Brazil

**Keywords:** cancer, drug delivery, human health, mimetics, nanotechnology

## Abstract

Biomimetic nanocarriers, engineered to mimic the characteristics of native cells, offer a revolutionary approach in the treatment of various complex human diseases. This strategy enhances drug delivery by leveraging the innate properties of cellular components, thereby improving biocompatibility and targeting specificity. Biomimetic nanocarriers demonstrate significant advancements in drug delivery systems against cancer therapy, Alzheimer's disease, autoimmune diseases, and viral infections such as COVID-19. Here, we address the therapeutic applications of biomimetic nanocarriers and their promising strategy for personalized medicine.

## Introduction

Human exposure to nanoparticles has naturally occurred for millennia, with a notable intensification following the industrial revolution [1]. The foundational concept of modern nanotechnology, introduced by Richard Feynman in 1959 during an American Physical Society meeting [1–2], involves the manipulation of matter at the atomic level. The term "nanometer" was initially proposed by Richard Zsigmondy in the context of measuring gold colloids. Nanotechnology is generally defined as the manipulation of matter on a nanoscale, typically ranging from 1 to 100 nm [2]. At this scale, nanoparticles can effectively interact with DNA and protein molecules [3–4].

**Figure 1:**
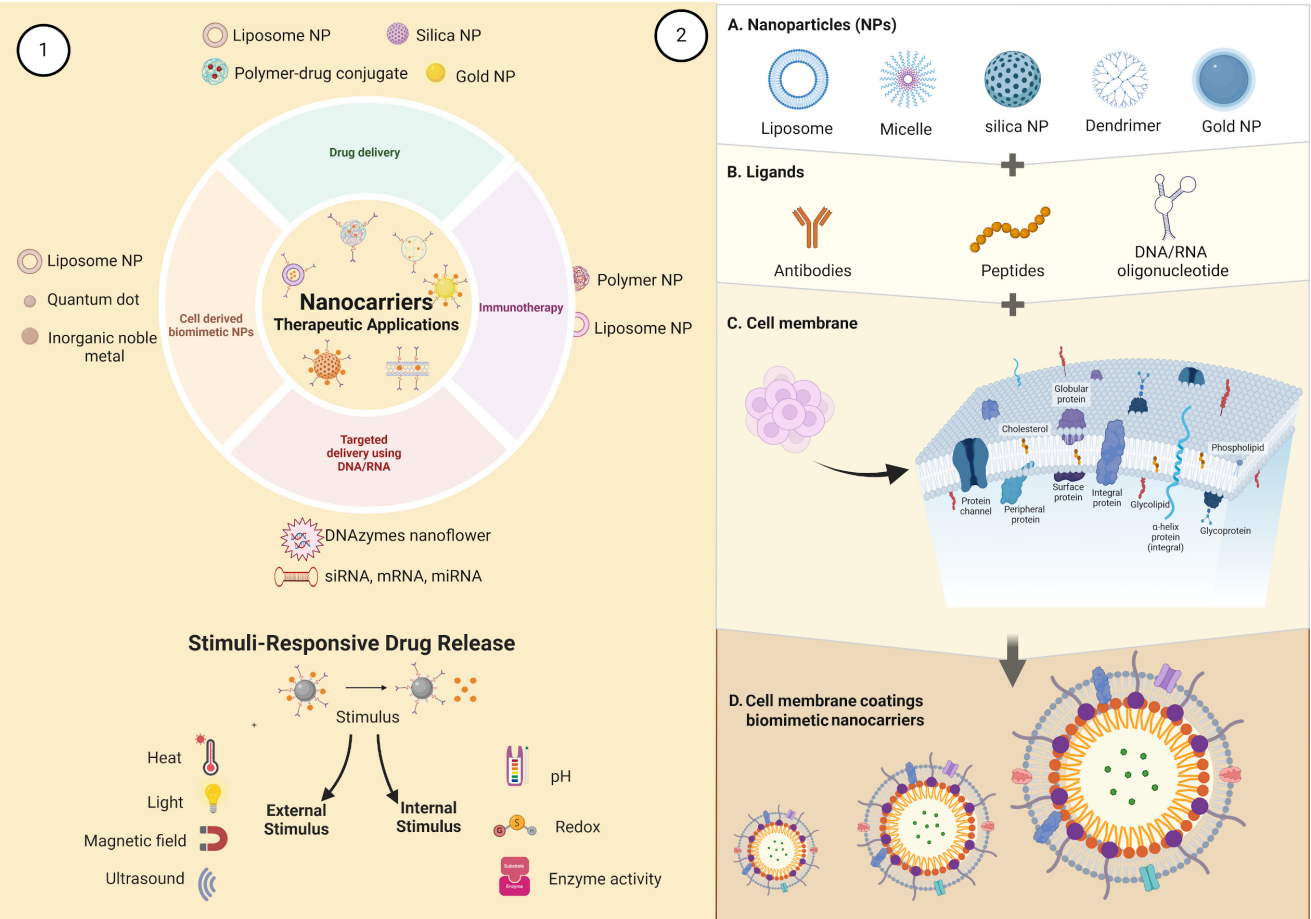
Schematic of biomimetic nanocarriers. Nanoparticles (NPs) used as nanocarriers in therapeutic applications such as drug delivery, immunotherapy, targeted delivery, biomimetic-derived NPs, and stimuli-responsive drug release (1). Coated NPs for improving biological activity through biomimetic biomembranes (2): Most used materials for creating nanoparticles (A), binders used for delivery and binding to target cells (B), characteristics of cell membranes (C), and biomimetic nanocarriers (D). Created in BioRender. Sodré, B. (2024) https://BioRender.com/n85g617. This content is not subject to CC BY 4.0.

Matter can exhibit distinct physical, chemical, and biological properties at the nanoscale compared to the macroscale, with significant differences in key characteristics. The National Nanotechnology Initiative (NNI) emphasizes that nanomaterials hold promising potential across various fields of knowledge [1,5]. Materials such as liposomes, nanoparticles, polymer–drug conjugates, inorganic noble metals, and quantum dots may improve therapeutical characteristics as demonstrated in Figure 1-1.

In the field of drug delivery, properties such as size, surface-to-volume ratio, and biocompatibility have driven the development of nanoscale-based devices [6–9]. Nanocompounds offer a strategic approach to addressing or at least improving the application of organic and inorganic compounds with activity against various diseases [10–12]. Faced with a physiological stimulus, the carrier decouples from the transported product and releases it at a specific interaction site (Figure 1-1). However, some challenges are encountered by these compounds such as: loss of stability, low efficiency in crossing biological barriers, inadequate efficacy in reaching target active molecules, and poor biodistribution [13–14]. Nanocarriers are employed to transport raw materials, which can be vesicles or solid nanoparticles [15]. Despite the significant advancements nanocarriers have brought to medical sciences, particularly in cancer treatment, several challenges remain for their widespread application. Issues such as cytotoxicity, difficulties in management, encapsulation, and in vivo release pose barriers to the application of nanocarriers [16–17].

In this context, biomimetic strategies using natural components emerge as revolutionary tools to overcome these challenges. The utilization of cellular components or parts thereof, such as macromolecules or membranes, can enhance drug delivery and therapeutic efficiency in the human body, representing a new opportunity for personalized therapies [12,18–19]. Here, we explored the implications of biomimetic nanostructured carriers and their applications in human health.

## Biomimetic Nanocarriers

**Figure 2:**
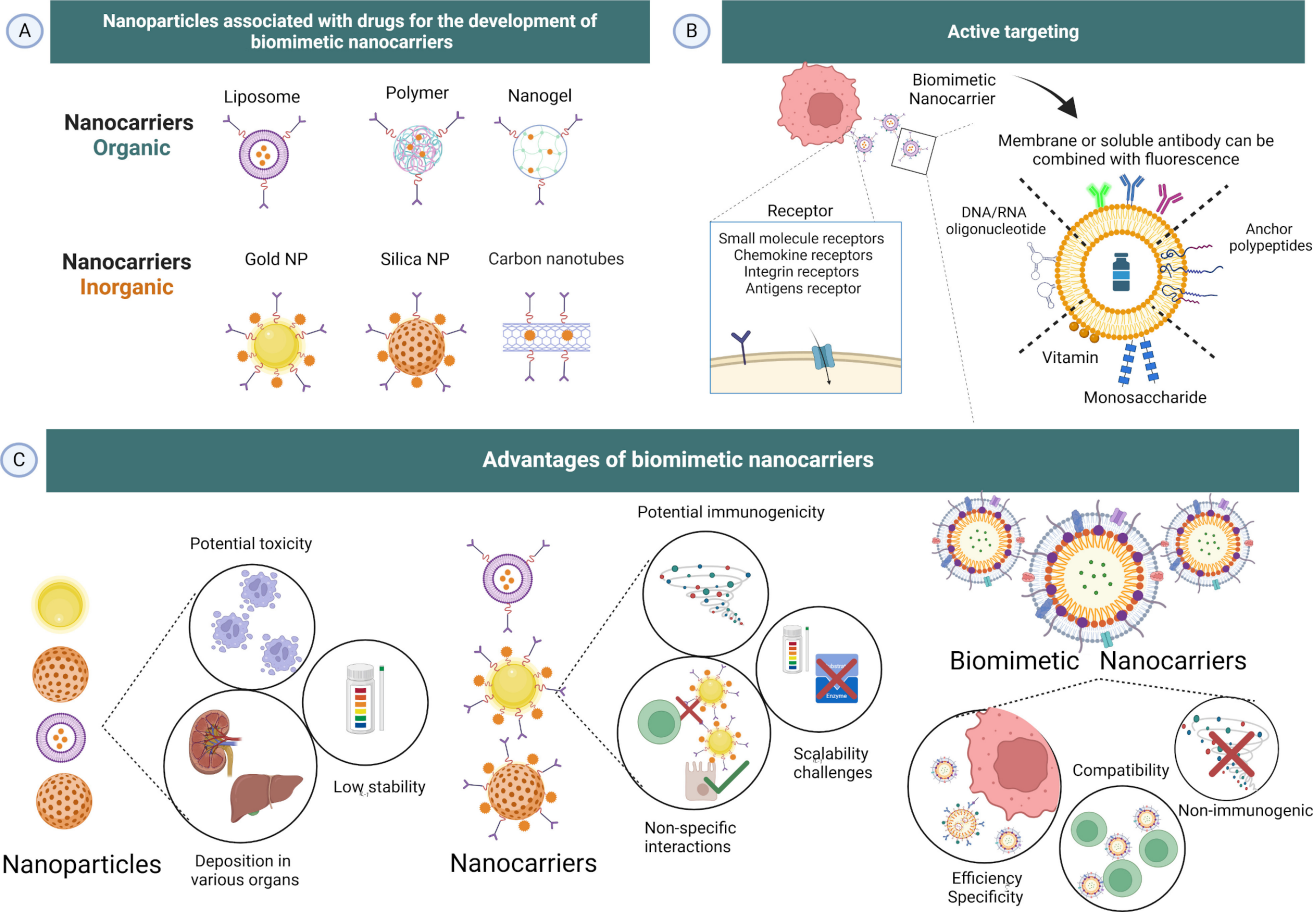
Action of biomimetic nanocarriers. Inorganic and organic nanoparticles with transport function (A); target with potential binding sites for drug delivery; (B) and coated nanocarriers improve functional activity and carrier efficiency (C). Created in BioRender. Sodré, B. (2024) https://BioRender.com/c20d446. This content is not subject to CC BY 4.0.

The principle of biomimetic nanocarriers involves coating nanoscale carriers with materials capable of replicating the characteristics or functions of native cells [19]. Nanoparticle coating involves obtaining nanoparticles (Figure 1-2A), which can be organic or inorganic in structure (Figure 2A), and conjugating them with functional ligands (Figure 1-2B) or biological structures, such as cell membranes (Figure 1-2C), which mask the nanocarriers and enhance biological activity (Figure 1-2D) [20]. This mimetic surface helps the device to mask epitopes potentially recognized by the immune system, thereby enhancing their biocompatibility. Additionally, the selectivity for targets and the circulation time of these carriers are improved [19,21–25]. Various cellular components such as extracellular vesicles, leukocyte and red blood cell membranes are beneficial for developing bioinspired devices. Specific targets, including peptides, aptamers, proteins, and viral capsids, may also be utilized in the production of nature-inspired synthetics as demonstrated in Figure 2B [22–26]. Indeed, the co-incubation of nanoparticles with cellular components creates an environment conducive to the absorption of proteins by the nanoparticles, thereby facilitating the connection of these structures [21–23].

The obtention of biomimetic nanocarriers can be achieved through two fundamental approaches: the synthetic engineering of biologically based components and the utilization of existing elements such as viral and bacterial vectors [27–29]. Drug delivery can occur through passive or active targeting mechanisms. In the passive strategy, coated nanocarriers can traverse permeable vessels (as observed in tumors, for example) and exhibit tropism toward specific pathological targets based on the size, surface charge, and physicochemical properties of the nanostructure. The active strategy involves surface coating with specific ligands which interact with elevated levels of target-specific receptors. Both delivery systems aim to achieve responsive drug release directly at the therapeutic target (Figure 2B) [19,30]. Therefore, a crucial step in constructing efficient biomimetic-based nanocarriers is understanding the fundamental building blocks, size, shape, and biological properties to mimic real cells and enable their internalization [31–32].

One efficient strategy for producing biomimetic nanocarriers involves camouflage with biological membranes. The phospholipids, anchored proteins, fatty acids, and other compounds present in these membranes not only confer cell-like properties to the carriers but also prevent immune recognition, extend circulation time, and enhance target mimicry, such as that of cancer cells [33]. Coating particles with membranes has been well-described for nanoparticles, and this process entails three steps: obtaining membrane-derived vesicles from a cellular source (1); generating the nanoparticles (2); and fusing the vesicles with the particles (3) [34–37].

Obtaining membrane vesicles requires the lysis of donor cells, necessitating an adequate number of cells [37]. Cells may be sourced from specific tissues or clonally expanded in the laboratory. Once sufficient cells are available, membrane vesicle isolation begins. Target cells are subjected to freeze–thaw cycles or hypotonic environments to induce cell lysis and release intracellular components [38–39]. The resulting product is then washed in a buffer solution containing protease inhibitors to eliminate cellular debris [40]. Subsequent sonication yields vesicles of 1 to 2 µm, and size homogenization can be achieved using a micro-extruder with a nanoscale membrane [41].

Once membrane vesicles are prepared, fusion with the nanocarrier can be accomplished by several methods [20,42]. Bath sonication disrupts membranes by forming cavitation bubbles, allowing them to reassemble around the nanocarrier. Optimizing this process requires adjusting exposure time, wave frequency, and temperature control [36,43]. However, due to vesicle fragmentation and reassembly, achieving uniform size can be challenging [44]. Electroporation involves exposing vesicles in a microfluidic device to an electric field, creating membrane pores for nanocarrier incorporation. Key parameters, including pulse voltage and exposure time, can be optimized to improve efficiency. Though costly, this method is suitable for industrial applications [34,45].

Another strategy exploits electrostatic charges of nanocarriers and membrane vesicles. Opposite charges (negative for vesicles and positive for carriers) foster electrostatic attraction, leading to spontaneous synthesis [46–48]. The process depends on electrostatic and hydrophobic interactions, where modulation of the carrier charge determines the strength of interaction and conjugation efficiency with the membrane vesicle [49–50]. A common technique for merging cellular membranes and carriers is coextrusion through polyester or polycarbonate membranes with various pore sizes [51–52]. In this method, mechanical extrusion forces the nanocarriers into the membrane vesicles. This approach yields product uniformity and preserves membrane protein layers, though it involves increased material waste and costs [34–35,53]. Regardless of the membrane-masking technique, various cell types can enhance the efficiency of delivery systems, including immune cells (phagocytes, lymphocytes, and NK cells) [54], erythrocytes [55], platelets [56], cancer cells [57] and hybrid membrane constructs [58].

Regarding materials for coating nanoparticles, a variety of hydrophilic polymers are available [30]. The most prevalent technique involves the use of polyethylene glycol (PEG). It provides biological protection against proteolysis and improved biocompatibility, metabolism, and drug absorption by the mononuclear phagocytic system due to its hydrophilic barrier [59]. Although PEG-coated nanostructures exhibit promising physicochemical properties, they have shown limitations; studies point to cases of hypersensitivity in PEGylated vaccines [60–61]. Potential adverse immune reactions have also been reported [59,61].

Nanocarriers have evolved into intricate chemical structures that include specific functionalities, allowing them to preferentially target sites of interest with their payload while minimizing immune clearance (Figure 2C) [62]. In studies on the anticancer activity of polylactic glycolic acid (PLGA) nanoparticles coated with membranes, Zhang et al. (2021) tested nanoparticles loaded with gambogic acid and coated with red-blood-cell-derived membranes in colorectal cancer cells. They demonstrated a reduction in phagocytosis, increasing the circulation time of the nanoparticles due to the coating [63]. A similar study with nanoparticles coated with cytotoxic T lymphocyte membranes for the treatment of gastric cancer showed a reduction in macrophage uptake compared to other membrane types [64]. Other studies on nanoparticles loaded with the antitumor molecule bufalin and covered with platelet membranes demonstrated their ability to evade macrophage uptake and enhance binding to target cancer cells. Together, these results confirm the ability of biomimetic coated nanostructures to evade the immune system, enabling prolonged circulation time and, consequently, sustained and controlled release of potential associated drugs [21].

To overcome these limitations and enhance coating efficiency, the decoration of nanostructures with functional ligands increases their biological interactions. Decreasing nonspecific interactions and immunogenicity is one of the main solutions that biomimetics addresses (Figure 2C). This approach has demonstrated that complex nanocarrier drug delivery systems need to exhibit compatible surfaces with target cells to enhance their functional capabilities [19].

## Biomimetic Nanocarriers in Human Health

The field of nanocarriers for drug delivery in cancer therapy has been extensively studied in the pursuit of biocompatible components with high specificity. In this context, the integration of synthetic compounds such as nanoparticles with natural components, including membranes from various cell types (e.g., erythrocytes, leukocytes, stem cells, tumor components) or other biocomponents (e.g., platelets), can enhance the functionality of carriers and meet the requirements for human applications [19].

The cellular membranes of cancer cells exhibit adhesion molecules crucial to cancer development and metastasis. Heterotypic or homotypic adhesive interactions through selectins, E-cadherins, Thomsen–Friedenreich (TF) antigens, the immunoglobulin superfamily (Ig-SF), and the interaction of SIRP-α with CD47 inhibit the phagocytosis of these cells, thus preventing their capture by dendritic cells [62,65–66]. Nanocarriers associated with cancer treatment offer numerous advantages, including immune evasion, targeting behavior, specific site accumulation, targeted delivery of drugs or genes, and reduced side effects. Studies involving inorganic nanocarriers with cell membrane coatings (CMC-NPs) have highlighted the importance of the homotypic behavior of CMC-NPs in delivering active therapeutic agents to specific sites, promoting immune evasion of CD47 cells by blocking binding with SIRP-α, preventing its phosphorylation, and thereby restoring the phagocytosis of cancer cells by macrophages. Additional studies have demonstrated that coated nanocarriers, such as PLGA NPs and silica NPs, enhance interactions with dendritic cells, leading to antitumor responses [25–26].

In a similar study with CMC-NPs composed of C-phycocyanin (C-PC) and a CD59-specific binding peptide (CD59sp), the antitumor activity of the C-PC/CMC-CD59sp nanoparticle was demonstrated by inhibiting proliferation through negative regulation of cyclin D1, halting the G0/G1 cell cycle in HeLa and SiHa cervical cancer cells [67–68]. Biomimetic-specific targets provide opportunities for personalized cancer therapies [10–12].

Coated nanocarriers have also been employed in treating other diseases, such as Alzheimer's disease. Current medications for Alzheimer's face the challenge of the blood–brain barrier (BBB), which includes the blood–brain, cerebrospinal fluid–brain, and blood–cerebrospinal fluid barriers. These barriers exhibit high selectivity in drug delivery due to their protective mechanism against harmful endogenous and exogenous substances through transcellular pathways. The BBB selectively permits molecules smaller than 400 Da with specific shapes, ionization states, and lipophilicity, thereby excluding macromolecular drugs and those with nonpermissive characteristics at the barrier [69].

Polymeric nanoparticles, as well as those based on lipids and inorganic materials, are extensively studied for Alzheimer's disease treatment due to their tissue selectivity, potential circulation time, encapsulation capacity, and, importantly, their ability to enhance BBB penetration. Studies have shown that polymeric biomimetic nanoparticles carrying proteins can penetrate the brain parenchyma and release active agents, demonstrated by the increased accumulation of 3D6-Fab antibody fragments in the brains of mouse models, and reducing Aβ1-42 aggregation, which is linked to dementia and neuronal loss [70].

Focusing on BBB compatibility, lipid-based nanoparticles demonstrate high potential in facilitating drug delivery. Macrophage membrane-coated liposomes co-modified with the RVG29 peptide and triphenylphosphine cation have shown improved targeting of brain neuronal mitochondria, as evidenced by fluorescence intensities identified in brain homogenates, and reduced Aβ1 deposition, demonstrating the ability of the nanocomposite to cross the BBB [71]. Inorganic nanoparticles exhibit unique optical, magnetic, and chemical properties and stability. Gold nanoparticles (AuNPs) with polyoxometalate and the peptides POMD and LPFFD (AuNPs@POMD-pep) have shown inhibition of Aβ1 aggregation and Aβ-induced cytotoxicity. However, the inherent toxicity of this formulation, challenges in particle digestion, and the potential for triggering immune reactions remain limiting factors [72–73].

Biomimetic nanocarriers coated with membranes from various cell types have also been applied in treating autoimmune diseases, such as rheumatoid arthritis, to optimize and enhance drug accumulation and delivery to specific sites. Studies with platelet membrane-coated nanoparticles loaded with the immunosuppressant FK506 have demonstrated increased delivery to inflammation sites and reduced symptoms such as redness and inflammation in the hind limbs of mice [27,74].

In the treatment of COVID-19, biomimetic nanocarriers have also been used to optimize anti-inflammatory and antiviral treatments. Tan et al. (2021) employed lopinavir (LPV), an antiviral drug, in polymeric nanoparticles coated with macrophage membranes (PLGA-LPV@M). This biomimetic nanocarrier demonstrated the ability to inherit the antigenic profile of macrophages, enabling the absorption of pro-inflammatory substances, increasing medication accumulation at the infection site, and reducing the adverse effects of the free medication [75]. Additionally, in tests with mice treated with PLGA-LPV@M, a 60% of improvement survival was observed compared to the control group (saline treated). Untreated animals rapidly lost weight and none survived for more than five days. This data demonstrates the effective therapeutic effect of biomimetic structures [75].

In addition to direct applications, interactions with the immune system targets also demonstrated the efficiency of biomimetic nanocarrier applications. To address the characteristic cytokine storm (as observed in COVID-19 disease), studies have developed nanocarriers containing squalene, adenosine, and vitamin E (SQAD/VitE) [76]. The anti-inflammatory properties of adenosine and the ability of squalene to prolong blood circulation time provide improved bioavailability and drug delivery. Additionally, the capacity of these nanocarriers to react with reactive oxygen species at inflammation sites offers an anti-inflammatory response, reducing tissue damage [76–77].

Within this scope, manganese dioxide nanocarriers have been studied to address a challenge in chemodynamical therapy (CDT): the high presence of reducing species (GSH) inhibit the therapeutic effects of CDT in tumors [78]. By releasing metal cations in the early stages to consume the reducing substances, these nanocarriers enhance therapeutic efficacy of CDT. This mechanism has also been applied to amphiphilic nanoparticles and ROS-responsive poly(α-l-lysine) nanoparticles, which are developed and used to encapsulate antibiotics, achieving both antibacterial and antioxidant functionalities [79–80].

## Conclusion

Biomimetic nanocarriers represent a promising strategy for the treatment of several clinically relevant and challenging human diseases. This study demonstrates that mimicking cell membranes, particularly those of immune system cells, offers significant benefits by reducing the degradation of biomaterials by the host. The use of biocompatible coatings not only enhances treatment efficacy but also paves the way for increasingly personalized therapeutic approaches. This strategy holds substantial potential for achieving high efficiency in treating diseases that currently lack a cure.

## Data Availability

Data sharing is not applicable as no new data was generated or analyzed in this study.
